# Evaluation of six different DNA extraction methods for detection of *Mycobacterium tuberculosis* by means of PCR-IS6110: preliminary study

**DOI:** 10.1186/1756-0500-6-561

**Published:** 2013-12-28

**Authors:** Isabela Neves de Almeida, Wânia da Silva Carvalho, Maria Lúcia Rossetti, Elis Regina Dalla Costa, Silvana Spindola de Miranda

**Affiliations:** 1 Federal University of Minas Gerais, Belo Horizonte, Brazil; 2 State Foundation for Production and Research in Health (FEPPS), Porto Alegre, Brazil; 3 Research Group Coordinator FM/UFMG_REDE-TB, Belo Horizonte, Minas Gerais, Brazil

**Keywords:** *M. tuberculosis*, IS6110, DNA extraction, PCR

## Abstract

**Background:**

Developments in molecular detection and strain differentiation of members of *Mycobacterium tuberculosis* complex have proved to be useful. The DNA extraction method influences the amplification efficiency, causing interference on the sensitivity and respective inhibitors. The aim of this study was to standardize a simple and fast DNA extraction method, providing DNA amplification by IS6110-PCR effectively free from undue interferences.

**Findings:**

The efficiency of the six different protocols tested in *M. tuberculosis* cultures has varied from 75% to 92.5%. This preliminary study evaluating the IS6110 PCR sensitivity and specificity was developed in DNA extracted from microscope slides, and achieved 100% of efficiency.

**Conclusions:**

DNA extraction by *Chelex + NP-40* method from both, cultures of *M. tuberculosis* and smear slides, resulted in good quantity of interference free DNA, especially in samples with low concentrations of genetic material; therefore, such technique may be used for the molecular diagnosis of tuberculosis.

## Background

Tuberculosis (TB) is one of the leading chronic bacterial infections, with mortality of almost 3 million and more than 8 million new cases every year [1]. Early diagnosis, effective treatment, and successful termination of transmission are major strategies for TB control [2].

Polymerase Chain Reaction (PCR) is a fast and sensitive diagnostic method for detection and identification of *M. tuberculosis*, especially in samples with poor load of bacilli [3]; and the most used molecular marker for DNA amplification is the IS6110 insertion element; which is specific for the genome of *M. tuberculosis* complex species [4].

Some studies report different sensitivity and specificity results, when using PCR techniques as diagnostic investigation tool; such results vary from 11 to 81% [5]. There are many reasons for this frequent variability of PCR results; however, earlier studies have suggested that PCR outcomes largely depend on the DNA extraction method [6].

DNA extraction methods should be effective, simple, and rapid, eliminating also the presence of PCR inhibitors during extraction [6,7].

Thus, the aim of this study was to standardize a simple, fast, and less complex method of DNA extraction, providing also DNA amplification by IS6110-PCR free from any undue interference.

## Methods

### Clinical data

The samples included in this study came from patients assisted in the Hospital of Clinics of the Federal University of Minas Gerais (UFMG), in the years of 2010 and 2011. Patients care comprised only individuals older than 18 years, who are presenting TB suspicion and have not started the treatment yet, as demonstrated in Tables 1 and 2. All patients that received TB diagnosis treated with anti-TB drugs.

**Table 1:** Clinical data from patients included in the protocols of extraction from cultures

**Patients**	**Gender**	**Origin**	**Hypothesis/Symptoms**	**Past TB**	**HIV**
3	m	Ward	Cough to clarify	No	Yes
4	m	Outpatient	TB	No	No
5	m	Ward	Fever to clarify	No	No
6	f	Ward	TB	No	No
7	m	EU	TB	No	No
8	m	Ward	TB	Yes	Yes
9	f	Ward	TB	No	No
10	m	Ward	TB	No	No

m = male; f = female; EU = emergency unit; TB = tuberculosis; HIV = *Human Immunodeficiency Virus.*

**Table 2:** Clinical data of patients included in protocols of extraction from microscope slides

**Patients**	**Gender**	**Origin**	**Hypothesis/Symptoms**	**Past TB**	**HIV**
1	m	Ward	Fever to clarify	No	Yes
2	f	Outpatient	Cough to clarify	No	No
3	m	Outpatient	TB	Yes	Yes
4	f	Outpatient	Cough and emaciation to clarify	No	No
5	f	Outpatient	TB or nocardiosis	Yes	No

m = male; f = female; TB = tuberculose tuberculosis; HIV = *Human Immunodeficiency Virus.*

### Samples

Ten bacterial dilutions of *M. tuberculosis* in Löwenstein-Jensen culture medium have been tested, using serial dilutions of 1/10, 1/100 and 1/1000; a total of 230 dilutions were tested with different DNA extraction methods.

Two bacterial suspensions of H37Rv strain of *M. tuberculosis* were used as positive control.

Five slides with positive bacilloscopy sputum (one + and two ++) and one with negative bacilloscopy were selected, coming from the Mycobacterial Laboratory of the Hospital of Clinics, at the Federal University of Minas Gerais. The slides were stained by fluorescence method (Auramine O), to be submitted to extraction procedure.

### Samples preparation

#### M. tuberculosis *inactivation*

For DNA extraction from culture, a bacterial suspension containing 1.5 mL of sterile water and around 3 to 5 colonies of H37Rv strain of *M. tuberculosis* was prepared in Eppendorf tubes. This suspension was inactivated at 100°C for 30 minutes in thermo block, and then centrifuged at 14,000 rpm for 10 minutes, at 4°C. The supernatant was discarded, and the sediment was used in Extraction Protocols 1, 2, 3, 4, and 5. For Extraction Protocol 6, the initial stage was different and will be described further.

### Preparation and staining of smears slides

The smears were done before the samples’ decontamination step with 0.5% *N*-acetyl-L-cysteine/2% NaOH (NaLC/NaOH) [8]. *M. tuberculosis* and Non-tuberculous Mycobacteria (NTM) species were confirmed after growth in Löwenstein-Jensen culture medium, and identified by basic biochemical methods, in the Ezequiel Dias Foundation Reference Center [9].

### Extraction

#### M. tuberculosis *culture DNA extraction*

### Extraction protocol 1 – extraction using phenol-chloroform

DNA from *M. tuberculosis* strains was prepared as follows, after neutralization: a pellet of 400 μL of lysozyme solution (10 mg/mL) was added to the suspension, and incubated for 1 hour at 37°C. Afterwards, 20 μL EDTA (50 mM) + 400 μL of proteinase K solution (10 mg/mL) were added, and the mixture was incubated at 60°C for 1 hour. Then, the solution was stored at -20°C overnight, and the DNA was extracted with phenol-chloroform and ethanol. After freezing, the solution was divided into two parts, and 400 μL of phenol-chloroform were added in each vortex tube and centrifuged at 12,000 rpm for 15 minutes. The supernatant was then transferred to another tube, and 0.6 volume of isopropanol and 1/10 volume of sodium acetate were added; homogenizing then the solution until white color disappearance. The solution was then stored again at -20°C, for 30 minutes. After that, it was centrifuged at 12,000 rpm for 10 minutes, and the supernatant was discarded. The pellet was washed twice with 500 μL of 70% ethanol, and after complete evaporation 20 μL of TE (Tris 100 μM + EDTA 50 μM) were added to it. The DNA was conserved at 2-8°C, until PCR development. This method was considered the standard method [10].

### Extraction protocol 2 – extraction using 70% alcohol

500 μL of 70% Ethanol were added to a pellet in a tube, and incubated for 2 hours. Then, mycobacterial cells were centrifuged at 13,000 rpm for 10 minutes, the supernatant was discarded, and the pellet was washed twice with sterile distilled water. After washing, the pellet was resuspended in 500 μL of sterile distilled water within an Eppendorf tube of 1.5 mL, being then used for PCR [11].

### Extraction protocol 3 – extraction using Chelex 100 + *Nonidet P-40 (NP-40)*

200 μl of solution were added to a pellet from Chelex suspension containing 5% Chelex-100, 1% Nonidet P-40, 1% Tween 20, and distilled water. After mixing thoroughly, the samples were maintained for 30 minutes at 100°C. The samples were then centrifuged for 10 minutes at 13,000 g, and the solution was transferred to a fresh microcentrifuge tube and used for PCR [8].

### Extraction protocol 4 – extraction using Chelex 100

200 μL of a solution containing 40 mg Chelex 100 + 100 mL of water were added to a pellet, and the resulting material was maintained at 95°C for 20 minutes. Then, it was centrifuged at 12,000 g for 15 minutes. The supernatant was used as a DNA source for PCR [12].

### Extraction protocol 5 – extraction using Chelex 100 + 70%Alcohol

A total of 150 μL of ice-cold 70% ethanol was added to a tube, mixed thoroughly, and maintained in ice (-20°C) for 20 minutes. The suspension was then centrifuged at 12,000 rpm for 5 minutes, and 200 μl of 20% Chelex solution were added to the pellet. The mixture was then vigorously stirred with a shaker, and incubated at 55°C for 1 hour. It was then placed in the vortex again, at high speed, for 10–20 seconds. The tubes were maintained at 100°C for 15 minutes. Then, the tubes were centrifuged at 12,000 rpm for 5 minutes, at 4°C. Afterwards, the supernatant was transferred to a new tube and used for PCR [6].

### Extraction protocol 6 – extraction using chloroform + CTAB*(N-cetyl-N,N,Ntrimethyl ammonium bromide)*

Several bacteria loopfuls were resuspended in 400 μL of TE 1X buffer, and then inactivated at 80°C for 20 minutes. 50 μL of lysozyme solution were added to the vortex and incubated at 37°C for at least 1 hour, under stirring. 70 μL of SDS 10% and 5 μL of proteinase K were added to the vortex, and incubated at 65°C for 10 minutes. 100 μL of NaCl 5 M and 100 μL of CTAB/NaCl solution were added to the vortex until the liquid content becoming white; then, it was incubated for 10 minutes at 65°C. 750 μL of chloroform/isoamyl alcohol (24:1) were added to the vortex for 10 seconds, and centrifuged at room temperature for 5 minutes, at 14,000 g. The supernatant was transferred to a clean tube and 0.6 volume of isopropanol was added. It was incubated at -20°C for 30 minutes, and centrifuged for 15 minutes at 14,000 g. The supernatant was discarded and the pellet washed with 1 mL of 70% ethanol and centrifuged for 5 minutes at 14,000 g. To precipitate the DNA, 20–30 μL of TE were added [13].

### *M. tuberculosis* DNA extraction from smear slides of sputum

#### Chelex 100 + Nonidet P-40 (NP-40) on slides

The sputum smears on slides were submitted to Chelex 100 *+* Nonidet P-40 (NP-40) extraction, as it was the most effective method among the six tested ones.

A volume of 25 μL of a suspension containing 5% Chelex-100, 1% Nonidet P-40, 1% Tween 20, and distilled water was spread on the smear slides with a pipette tip. The liquid was transferred to an Eppendorf tube and 75 μL of the same suspension were added. After mixing thoroughly, the samples were incubated for 30 minutes at 100°C. The samples were then centrifuged for 10 minutes at 13,000 g, and the solution was transferred to a fresh microcentrifuge tube; then, 5 μL were used for PCR [8].

#### PCR and electrophoresis

PCR was performed in a final volume of 50 μL containing 7.0 μL of Buffer (10x), 3.0 μL of MgCl_2_ (50 mM), 0.2 μL of DNTP (25 mM), 25 pmol of each oligonucleotide (IS1- 5′ CCT GCG AGC GTA GGC GTC GG 3′ and IS2- 5′ CTC GTC CAG CGC CGC TTC GG 3′) and 0.5 μL of Taq DNA Polymerase (500 U) Invitrogen®. Amplification was carried out for 40 cycles, each consisting of initial denaturation at 94°C for 2 min, denaturation at 94°C for 30 seconds, annealing at 64°C for 2 minutes, extension at 71°C for 1 minute, followed by a final extension at 72°C for 10 minutes. PCR products were analyzed by gel electrophoresis in 2% agarose gel. Target DNA fragments of one hundred twenty-three base pairs (bp) were viewed under UV light.

#### Quantitative assessment of extracted DNA

The DNA dosing was executed by spectrophotometry (SpectraMax Plus®). The absorbance and DNA concentration (ideal 5–100 ng/μL) were evaluated as indicative of nucleic acids purity (ideal ratios: A_260_/A_280_ ≥ 1. 8 and A_260_/A_230_ = 2) [14,15].

A volume of 5 μL of concentrated DNA, and the respective dilutions (1/10, 1/100, and 1/1000), were submitted to PCR reaction, and dosed in the spectrophotometer SpectraMax Plus® (using 2 μL as per manufacturer’s instruction).

## Findings

**Figure 1:**
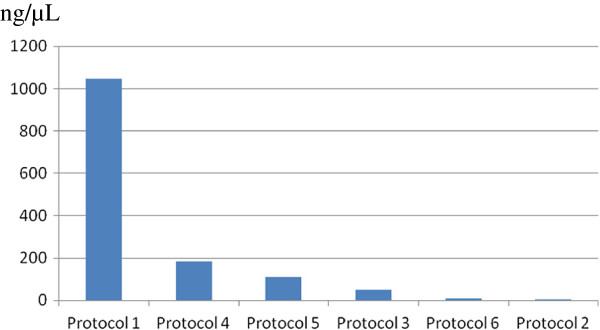
**Dosages of the extracted DNA using six different methods.** Concentrated DNA. “blue square”.

*M. tuberculosis* DNA dosing exhibited concentrations ranging from 1.048 ng/μL; 4.33 ng/μL; 47.78 ng/μL; 182.5 ng/μL; 112.3 ng/μL; 7.0 ng/μL in protocols 1, 2, 3, 4, 5, and 6, respectively (Figure 1).

The average value of A_260_/A_280_ and A_260_/A_230_ ratios is demonstrated in Table 3.

**Table 3:** Average absorbance with extraction protocols

	**A**_**260**_**/A**_**280**_	**A**_**260**_**/A**_**230**_
	**(≥1.8)**	**(<2.0)**
Protocol 1	1.96	1.69
Protocol 2	1.24	-0.74
Protocol 3	1.28	0.62
Protocol 4	0.46	0.13
Protocol 5	1.26	0.46
Protocol 6	-1.13	0.21

The efficiency of Extraction Protocols 1, 2, 3, 4, 5 and 6, developed in *M. tuberculosis*.

Cultures was respectively of: 75% (30/40), 75% (30/40), 90% (36/40), 75% (30/40) 77.5% (31/40), and 92.5% (37/40).

The methods presenting better performance were Extraction Protocols 3 and 6 (90% and 92.5%); however, protocol 3 did not show negative results even in dilution 1/1000.

The amplification results after DNA extraction from ten *M. tuberculosis* cultures are shown in Table 4.

**Table 4:** **Amplification after extraction of ten *M. tuberculosis *** **cultures**

		**DNA of *tuberculosis *** **cultures**									
		**1 (PC)**	**2**	**3**	**4**	**5**	**6**	**7**	**8**	**9**	**10**
Protocol	Concentrated	+	+	+	-	+	+	+	+	+	+
1	Dilution 1:10	+	+	+	-	+	+	+	-	+	+
	Dilution 1:100	-	+	+	-	+	+	+	+	+	-
	Dilution 1:1000	-	+	-	+	+	+	-	+	+	-
Protocol	Concentrated	+	+	+	-	+	+	+	+	+	+
2	Dilution 1:10	+	-	+	-	+	+	+	+	-	+
	Dilution1:100	+	-	+	+	+	+	+	+	-	+
	Dilution 1:1000	+	-	+	+	+	+	-	+	-	-
Protocol	Concentrated	+	+	+	+	+	+	+	+	-	+
3	Dilution 1:10	+	+	+	+	+	+	+	+	+	+
	Dilution 1:100	+	+	+	-	+	+	+	+	-	+
	Dilution 1:1000	+	+	+	-	+	+	+	+	+	+
Protocol	Concentrated	+	+	+	+	+	+	+	+	-	+
4	Dilution 1:10	+	+	+	-	+	+	+	+	-	+
	Dilution 1:100	+	+	+	-	-	+	+	+	-	+
	Dilution 1:1000	+	-	+	-	-	+	+	-	+	+
Protocol	Concentrated	+	+	+	+	+	+	+	+	+	+
5	Dilution 1:10	+	+	+	+	+	+	+	+	+	+
	Dilution 1:100	+	+	+	-	+	+	+	+	-	+
	Dilution 1:1000	+	-	-	-	+	+	-	-	-	-
Protocol	Concentrated	+	+	+	-	+	+	+	+	+	+
6	Dilution 1:10	+	+	+	+	+	+	+	+	+	+
	Dilution 1:100	+	+	+	+	+	+	+	+	+	+
	Dilution 1:1000	+	+	+	-	-	+	-	+	+	+

PC: positive control; + positive; - negative.

**Figure 2:**
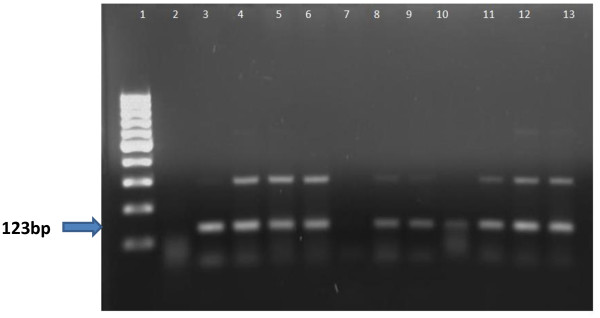
**Agarose gel electrophoresis of amplifications performed with protocol 3.** Lane 1 = Molecular marker 100 bp, Lane 2 = Negative Control, Lane 3 = Positive Control, Lane 4 = Sample 8, Lane 5 = Sample 6, Lane 6 = Sample 7, Lane 7 = Sample9, Lane 8 = Sample 5, Lane 9 = Sample 10, Lane 10 = Sample 4, Lane 11 = Sample 3, Lane 12 = Sample 1, Lane 13 = Sample 2.

Figure 2 shows electrophoresis of amplifications performed with Extraction Protocol 3.

**Figure 3:**
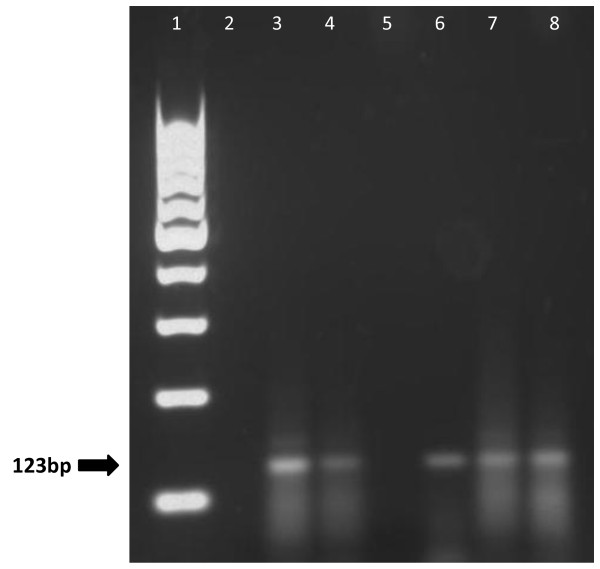
**Electrophoresis of amplifications of smear slides.** Lane 1 = Molecular marker 100 bp, Lane 2 = Negative Control, Lane 3 = Positive Control, Lane 4 = Sample 1, Lane 5 = Sample 5, Lane 6 = Sample 3, Lane 7 = Sample 3, Lane 8 = Sample 4.

There was amplification in 4 slides with *M. tuberculosis* positive cultures, and the slide with *M. kansasii* culture was not amplified by IS6110-PCR. The DNA extracted from the smear slides using Extraction Protocol 3 are shown in Table 5. Electrophoresis from smear slides amplification is shown in Figure 3.

**Table 5:** Results of amplifications after extraction of DNA from five smears in sputum slides

	**AFB AU**	**PCR**	**Culture**
Sample 1	++	pos	*M. tuberculosis*
Sample 2	+	pos	*M. tuberculosis*
Sample 3	-	pos	*M. tuberculosis*
Sample 4	++	pos	*M. tuberculosis*
Sample 5	+	neg	*M. kansasii*

+ and ++ positives; pos = positive; neg = negative; PCR = Polymerase Chain Reaction; AFB AU = Acid-fast bacilli by Auramine.

## Discussion

DNA dosing from cultures after extraction is an important parameter to quantify the extracted amount and evaluate its quality (purity); however, as evidenced in our results, it is not an absolute parameter [16,17], as large variation was verified either in ‘DNA concentration’ or ‘absorbance’ values, with no relationship with PCR-IS6110 amplification.

This study demonstrated the application of several DNA extraction methods, and found an inter-methods efficiency variation of 75% to 92.5% of PCR-IS6110, which proves that the extraction method does not exert direct influence on the IS6110-PCR effectiveness.

The number of steps and reagents, and the nature of chemical reagents (proteolytic enzymes, organic solvents, alcohols, and resins) used show the difference between the six extraction methods. Extraction Protocols 1 and 6 (*Phenol-chloroform and Chloroform + CTAB*) used lysozyme and proteinase K in the first extraction phase, which led to cellular membranes rupture and release of cytoplasmic components, due to digestion by proteolytic enzymes. Organic solvents as phenol and chloroform were also used, separating DNA from lipids and other biochemical compounds; the addition of ethanol occurred as well, to recover and purify the DNA. The use of CTAB allowed to improve the sensitivity, as it has better action on DNA purification [14].

Extraction Protocols 2 and 5 (*70% Alcohol* and *Chelex 100 + 70% Alcohol*) used 70% alcohol aiming DNA purification. 70% alcohol helps in the removal of organic waste that could act as PCR inhibitors [10]. Within these two protocols, elimination of large amounts of organic waste in the concentrated samples is evidenced through the action of 70% alcohol, which is best shown in Extraction Protocol 5, as all concentrated cultures were amplified, and resin (Chelex 100) was used to help the DNA removal from cell inside [6].

Extraction Protocols 3 and 4 (*Chelex + NP-40* and *Chelex 100*) used Chelex 100 resin as principal reagent for DNA extraction, which is associated to thermal shock and NP-40 in order to purify the DNA. These extraction protocols are fast; however, Extraction Protocol 3, which uses NP-40, had a higher efficiency when DNA was amplified (90%), demonstrating its possible use for saving time as compared to Extraction Protocol 1 (Phenol Chloroform), which has been described by some authors as the gold standard for DNA extraction [18].

Extraction Protocol 3 presented the best culture results in extraction; so, using this protocol in smear slides enables the recovery of smaller amounts (dilution 1/1000) of *M. tuberculosis* DNA, consuming less reagents, with less stages, and without inhibitors; so, this method is effective, practical, and fast.

In this work, we have noticed that a simpler protocol (Extraction Protocol 3), presenting fewer stages and consuming less reagents, presented results similar to those from more complex protocols (Extraction Protocol 6), presenting also higher efficiency with lower DNA concentrations (1/1000). Protocol 3 has been employed by some authors, since it does not use any organic solvent, eliminates multiple stages of purification, and uses only two Eppendorf tubes per sample, decreasing so the costs and time spent [7].

The authors used Extraction Protocol 3 (*Chelex + NP-40*) for DNA extraction in smear slides, due to its higher efficiency between protocols tested in culture. The good PCR performance exhibited in samples extracted by *Chelex + NP-40* may be associated to the fact that NP-40 is a detergent with high capacity for breaking lipid-protein interactions, performing so the cells lysis and facilitating DNA release from them; which results in good extraction and reduces the presence of PCR inhibitors. In addition, it is a single stage extraction method, eliminating so the DNA loss that occurs when using multiple stages.

This is a preliminary study with low number of samples; however, as molecular tests are very expensive in developing countries and, therefore, not routinely required as diagnostic tools by physicians, the implantation of these protocols in such countries could be a valid alternative to investigate TB; in addition, the cases included in this study are suspected of having TB and, effectively, they proceed from a high complexity hospital of the public health system.

The limitation of smear slides requires the use of a large panel in the study, to confirm sensitivity and specificity values; so, Extraction Protocol 3 would be ideal for laboratories where only bacilloscopy is performed, as is the case of several locations in Brazil. Additionally, it would eliminate the ‘biosafety issue’ during transportation of slides to molecular biology labs. The relevance of amplifying DNA extracted directly from smear slides is because bacilloscopy does not differentiate bacterial species, while IS6110 sequence amplification allows a rapid identification of these mycobacteria, as it is a specific sequence of MTC, distinguishing them from MNT. Therefore, new “in house” extraction methods should be standardized and tested, as shown in this study.

## Conclusion

The results of this work lead to conclusion that *Chelex + NP-40* method (Extraction Protocol 3) for *M. tuberculosis* DNA extraction from cultures and slides, is able to provide a good quantity of interference free DNA, mainly in samples with low concentrations of genetic material; which justifies its use in the molecular diagnosis of TB.

## Abbreviations

M. tuberculosis: *Mycobacterium tuberculosis*; TB: Tuberculosis; MTC: *Mycobacterium tuberculosis* complex; PCR: Polimerase chain reaction.

## Competing interests

The authors declare no competing interests.

## Authors’ contributions

SSM, WSC and INA planned and designed the experiments. INA performed the experiments. SSM, WSC and INA analyzed the data. SSM, WSC, INA, MLR and ERDC wrote the paper. All authors read and approved the final manuscript.
